# Gas-Phase Infrared Action Spectroscopy of CH_2_Cl^+^ and CH_3_ClH^+^: Likely Protagonists in Chlorine Astrochemistry

**DOI:** 10.3390/molecules29030665

**Published:** 2024-01-31

**Authors:** Sven Thorwirth, Kim Steenbakkers, Timon Danowski, Philipp C. Schmid, Luis Bonah, Oskar Asvany, Sandra Brünken, Stephan Schlemmer

**Affiliations:** 1 I. Physikalisches Institut, Universität zu Köln Zülpicher, Str. 77, 50937 Köln, Germany; tdanowsk@smail.uni-koeln.de (T.D.); schmid@ph1.uni-koeln.de (P.C.S.); bonah@ph1.uni-koeln.de (L.B.); asvany@ph1.uni-koeln.de (O.A.); schlemmer@ph1.uni-koeln.de (S.S.); 2 FELIX Laboratory, Institute for Molecules and Materials, Radboud University, Toernooiveld 7, 6525 ED Nijmegen, The Netherlands; kim.steenbakkers@ru.nl (K.S.); sandra.bruenken@ru.nl (S.B.)

**Keywords:** chlorine chemistry, astrochemistry, vibrational spectroscopy, ion trap, action spectroscopy

## Abstract

Two fundamental halocarbon ions, CH_2_Cl^+^ and CH_3_ClH^+^, were studied in the gas phase using the FELion 22-pole ion trap apparatus and the Free Electron Laser for Infrared eXperiments (FELIX) at Radboud University, Nijmegen (the Netherlands). The vibrational bands of a total of four isotopologs, CH_2_^35,37^Cl^+^ and CH_3_^35,37^ClH^+^, were observed in selected wavenumber regions between 500 and 2900 cm^−1^ and then spectroscopically assigned based on the results of anharmonic force field calculations performed at the CCSD(T) level of theory. As the infrared photodissociation spectroscopy scheme employed probes singly Ne-tagged weakly bound complexes, complementary quantum-chemical calculations of selected species were also performed. The impact of tagging on the vibrational spectra of CH_2_Cl^+^ and CH_3_ClH^+^ is found to be virtually negligible for most bands; for CH_3_ClH^+^–Ne, the observations suggest a proton-bound structural arrangement. The experimental band positions as well as the best estimate rotational molecular parameters given in this work provide a solid basis for future spectroscopic studies at high spectral resolutions.

## 1. Introduction

Out of the more than 300 inter- and circumstellar molecules detected to date, more than 25% contain heavy elements from the second row of the periodic table. With nearly fifty species, molecules containing silicon and sulfur are particularly abundant (an up-to-date list of astronomically detected molecules with complementary information is maintained at the Cologne Database for Molecular Spectroscopy (CDMS) [1,2] accessible online at https://cdms.astro.uni-koeln.de/ (accessed on 12 December 2023)).

In contrast, the number of chlorine-bearing molecules is much smaller. Only seven such species have been observed so far: HCl [3]; AlCl, NaCl, and KCl [4]; HCl^+^ [5]; H_2_Cl^+^ [6]; and CH_3_Cl [7]. The latter, pentatomic methyl chloride, or CH_3_Cl, is the most complex chlorine-bearing species presently known in space and, thus, an interesting target for studies of chlorine astrochemistry. In this context, it is assumed that CH_3_Cl may be formed through reactions on grain surfaces but also possibly in the gas phase through ion–molecule reactions, for example, in a scenario suggested by Garrod (cf., Ref. [8]).

(1) $${{\mathrm{CH}}_{3}}^{+}\;+\;\mathrm{HCl}\;\to \;{\mathrm{CH}}_{2}{\mathrm{Cl}}^{+}\;+\;H_{2}$$

(2) $${\mathrm{CH}}_{2}{\mathrm{Cl}}^{+}\;+\;H_{2}\;\to \;{\mathrm{CH}}_{3}{\mathrm{ClH}}^{+}\;+\;h\nu$$

(3) $${\mathrm{CH}}_{3}{\mathrm{ClH}}^{+}\;+\;e^{-}\;\to \;{\mathrm{CH}}_{3}{\mathrm{Cl}}^{+}\;+\;H$$

Here, in the first step, the chloromethyl ion CH_2_Cl^+^ is formed from a reaction of the methyl cation CH_3_^+^ and HCl; in the second, the halonium ion CH_3_ClH^+^ is produced via radiative association between CH_2_Cl^+^ and molecular hydrogen, finally yielding CH_3_Cl through dissociate recombination with a free electron.

While the CH_3_^+^, HCl, H_2_, and CH_3_Cl species are all known to be present in astronomical environments (the non-polar CH_3_^+^ molecular ion being detected only very recently through observations with the James Webb Space Telescope [9]), our knowledge of the CH_2_Cl^+^ and CH_3_ClH^+^ molecular ions also participating in the above chain of reactions is still highly fragmentary. From the viewpoint of laboratory experiments, only little spectroscopic information has been collected previously for the chloromethyl cation. Photoelectron spectroscopy of the CH_2_Cl radical has provided a vibrational wavenumber ν=1040(30) cm^−1^ of the Cl–C-stretching mode of CH_2_Cl^+^ [10,11]. A somewhat more comprehensive picture of the vibrational spectrum has been obtained through infrared spectroscopy of CH_2_Cl^+^ trapped in solid Ar matrices [12]. In these studies, four out of the six vibrational fundamentals were observed for the most abundant isotopic species, CH_2_^35^Cl^+^. Using precursors enriched in ^13^C or deuterium permitted the observation of four vibrational fundamentals of ^13^CH_2_^35^Cl^+^ or five of CD_2_^35^Cl^+^, respectively. Owing to a significant isotopic shift, the C–^37^Cl stretching mode could also be observed for all three isotopic species. Direct infrared spectroscopic studies of CH_2_Cl^+^ in the gas phase have not been reported yet. Hexatomic protonated methyl chloride, CH_3_ClH^+^, has been known from mass spectrometry and studies of reaction kinetics [13,14]; however, it has never been spectroscopically studied.

In the present work, the vibrational spectra of the ^35^Cl and ^37^Cl isotopic species of CH_2_Cl^+^ and CH_3_ClH^+^ were observed in the gas phase, marking the first direct gas-phase infrared study of CH_2_Cl^+^ and the first ever spectroscopic detection of CH_3_ClH^+^. The characterization of both ions was accomplished in an ion trap using a messenger action spectroscopy scheme, i.e., employing infrared photodissociation (IRPD) of their weakly bound complexes with neon, CH_2_Cl^+^-Ne, and CH_3_ClH^+^-Ne. Spectroscopic analysis was supported based on complementary high-level quantum-chemical calculations performed at the CCSD(T) level of theory. A detailed account of the experimental and theoretical work as well as of the analysis will be given in the following.

## 2. Results and Discussion

### 2.1. Structures of CH_2_Cl^+^, CH_3_ClH^+^, and Their Weakly Bound Complexes with Ne

**Figure 1:**
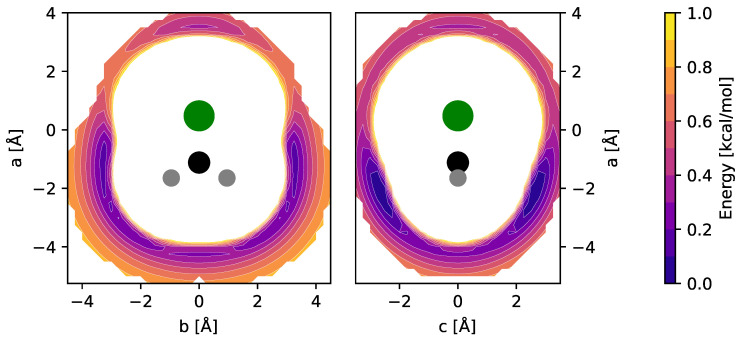
fc-CCSD(T)/aug-cc-pV(T + *d*)Z potential energy maps of the CH_2_Cl^+^–Ne weakly bound complex calculated in the two mirror planes of CH_2_Cl^+^ (see text for details). Atom color code: hydrogen (gray), carbon (black), chlorine (green). Contours show the potential energy of the complex as a function of the Ne atom position relative to the ion and cover the interval [0.1, 1.0] kcal/mol in steps of 0.1 kcal/mol above the global minimum.

Equilibrium molecular structures of CH_2_Cl^+^ and CH_3_ClH^+^ (and their isoelectronic sulfur-bearing counterparts, cf. Section 4) were calculated at the CCSD(T) level of theory using different basis sets. Full sets of internal coordinates are given in Appendix A and Appendix B. Best-estimate equilibrium structures were calculated using the cc-pwCVQZ basis set and all electrons in the correlation treatment, providing a level of theory previously shown to be of high accuracy [15]. The fc-CCSD(T)/cc-pV(T + *d*)Z level was employed for the calculation of harmonic and anharmonic force fields of the bare ions and used for the interpretation of their vibrational spectra. The impact of Ne-tagging on the molecular structures and harmonic force fields was estimated at the fc-CCSD(T)/aug-cc-pV(T + *d*)Z level of theory. To determine the energetically favorable locations of the Ne atom with respect to the CH_2_Cl^+^ and CH_3_ClH^+^ ions, potential energy surfaces (PESs) were calculated using grids of appropriate size and sampling (see also Ref. [16]). Briefly, in these calculations, the fc-CCSD(T)/aug-cc-pV(T + *d*)Z equilibrium structures of CH_2_Cl^+^ and CH_3_ClH^+^ were kept fixed while varying the position of the Ne atom on the grid over an area of a few tens of Å^2^ and at a spacing of 0.25 Å. At each of the grid points, single-point energy calculations were performed. PESs were calculated for the two mirror planes of CH_2_Cl^+^ and for the single mirror plane of CH_3_ClH^+^. The resulting potential energy maps of CH_2_Cl^+^ are shown in Figure 1.

**Figure 2:**
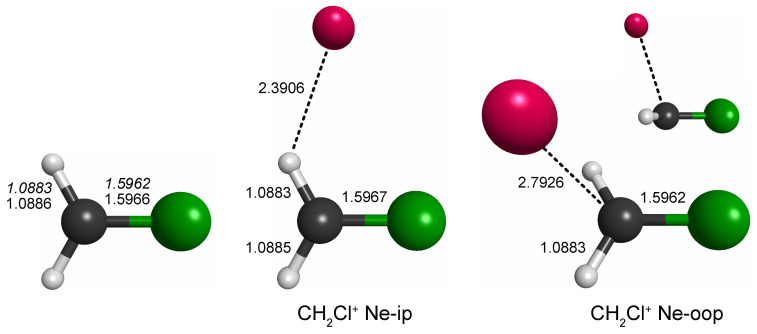
Bond lengths of CH_2_Cl^+^ and its complexes with neon (in Å; fc-CCSD(T)/aug-cc-pV(T + *d*)Z values of all three species given in regular font and fc-CCSD(T)/cc-pV(T + *d*)Z values of the bare species given in italics). Bare CH_2_Cl^+^ is of $C_{2v}$ symmetry, whereas the symmetry of the Ne-complexes is lowered to $C_{s}$. Full sets of structural parameters are given in Appendix A. For further details, see text.

Out of the four unique local minima seen, the shallow ones corresponding to linear C–Cl–Ne and Cl–C–Ne cluster variants were not considered further. The global minimum arrangements of each plot, i.e., one H-bound in-plane variant (CH_2_Cl^+^ Ne-ip, Figure 1, left map) and the out-of-plane variant (CH_2_Cl^+^ Ne-oop, right), were finally structurally refined in fully relaxed calculations. The corresponding structures are shown in Figure 2, and the full sets of structural parameters are given in Appendix A. As can be seen, the molecular structure of CH_2_Cl^+^ hardly changes upon Ne-tagging at either position, and as a consequence, also the harmonic vibrational wavenumbers of CH_2_Cl^+^ collected in Table 1 show very little dependence on tagging. The Ne-ip and Ne-oop species are calculated to be almost isoenergetic, with the latter configuration being slightly more stable by about 0.1 kcal/mol. Ne bond dissociation energies under the consideration of an estimate of zero-point vibrational effects amount to 0.6 kcal/mol (Ne-ip) and 0.7 kcal/mol (Ne-oop), respectively.

**Figure 3:**
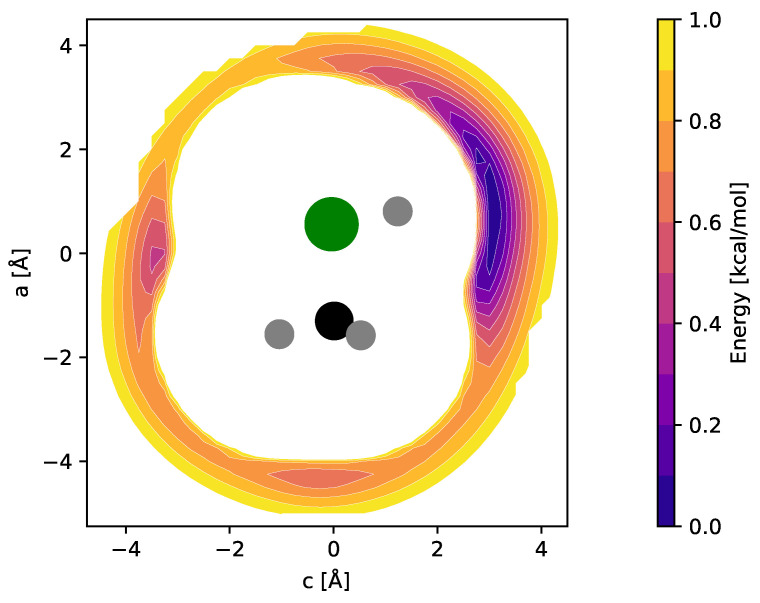
fc-CCSD(T)/aug-cc-pV(T + *d*)Z potential energy map of the CH_3_ClH^+^–Ne weakly bound complex calculated in the mirror plane of CH_3_ClH^+^ (see text for details). Atom color code: hydrogen (gray), carbon (black), chlorine (green). Contours show the potential energy of the complex as a function of the Ne atom position relative to the ion and cover the interval [0.1, 1.0] kcal/mol in steps of 0.1 kcal/mol above the global minimum.

**Figure 4:**
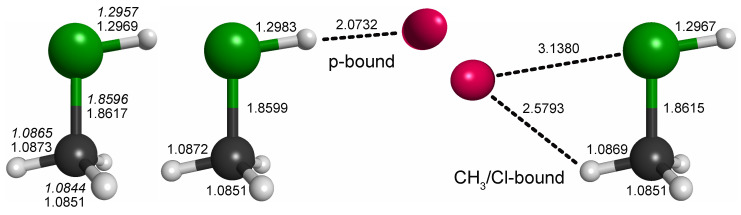
Bond lengths of CH_3_ClH^+^ and the p-bound and CH_3_-bound complexes with neon (in Å; fc-CCSD(T)/aug-cc-pV(T + *d*)Z values of all three species given in regular font and fc-CCSD(T)/cc-pV(T + *d*)Z values of the bare species given in italics). All species exhibit $C_{s}$ symmetry. Full sets of structural parameters are given in Appendix A. For further details, see text.

For CH_3_ClH^+^, by analogy with other rare gas-tagged protonated species, one would expect the Ne atom to be bound to the proton in a near-linear Cl-H⋯Ne fashion (e.g., Refs. [17,18,19]). Indeed, from the potential energy map obtained for the molecular mirror plane (Figure 3), this arrangement is identified as a deep, putatively global minimum. Another minimum is identified on the opposite side of the molecule and a third shallow one where Ne is arranged in a near-linear Cl-C-Ne fashion. Fully relaxed structures of the first two configurations (“p-bound” and “CH_3_/Cl-bound”) are shown in Figure 4. According to those calculations, the p-bound variant is more stable by 0.4 kcal/mol. The third minimum as well as potential arrangements where the Ne atom would be located outside of the symmetry plane of CH_3_ClH^+^ have not been studied further. The results of harmonic force field calculations to study the influence of Ne-tagging are presented in Table 2. As can be seen, the influence of tagging is virtually negligible for the majority, but not all, of the vibrational fundamentals (see further discussion in Section 2.3). Under the consideration of zero-point vibrational effects, the Ne bond dissociation energies are 1.0  kcal/mol and 0.7  kcal/mol for the p-bound and CH_3_/Cl-bound species, respectively.

### 2.2. IRPD Spectrum of CH_2_^35^Cl^+^–Ne

**Figure 5:**
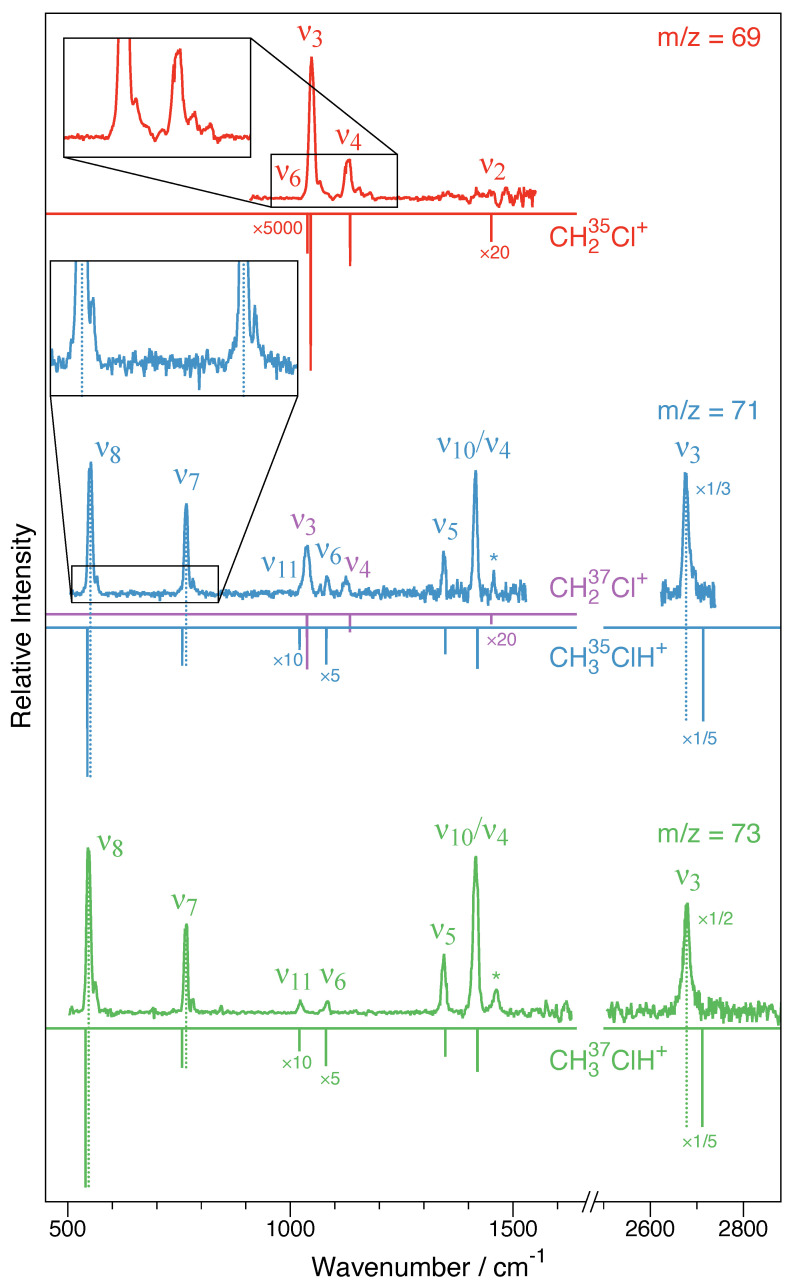
CH_2_Cl^+^–Ne and CH_3_ClH^+^–Ne infrared photodissociation (IRPD) spectra as obtained here. The traces indicate m/z=69 corresponding to the CH_2_^35^Cl^+^–Ne species (top spectrum in red), m/z=71 (isobaric mixture of CH_2_^37^Cl^+^–Ne and CH_3_^35^ClH^+^–Ne, blue center trace) and m/z=73 (CH_3_^37^ClH^+^–Ne, green bottom trace). For comparison, the vibrational fundamentals from VPT2 calculations (fc-CCSD(T)/cc-pV(T + *d*)Z) of the bare ions are shown as inverted sticks. For clarity, the intensities of selected calculated and experimental bands have been multiplied by the factors indicated. For the CH_3_ClH^+^–Ne species, dashed sticks indicate blueshifts of the $\nu_{8}$ and $\nu_{7}$ bands and a redshift of the $\nu_{3}$ band, which is indicative of Ne being bound to the proton in a near-linear Cl-H⋯Ne fashion. The bands labeled with an asterisk are not vibrational fundamentals. Insets highlight blueshifted satellites, presumably due to combination modes involving the Ne tag. For further details, see text.

The m/z=69 filtered IRPD spectrum from 900 to 1600 cm^−1^ is shown in Figure 5 (top red trace). From Table 3, it can be seen that four fundamental modes of the CH_2_^35^Cl^+^ species are calculated in this wavenumber range: the $\nu_{6}$ mode at 1038 cm^−1^, $\nu_{3}$ at 1046 cm^−1^, $\nu_{4}$ at 1134 cm^−1^, and $\nu_{2}$ at 1451 cm^−1^, only two of which have sizable calculated infrared intensities: $\nu_{3}$ (Cl–C stretching) and $\nu_{4}$ (out-of-plane (oop) bending). While infrared intensities are not strictly related to the intensities in the action spectroscopy scheme employed here, usually good qualitative agreement is observed. Clearly, $\nu_{3}$ and $\nu_{4}$ are detected at 1048 cm^−1^ and 1129 cm^−1^, respectively, which is in very good agreement with the anharmonic CCSD(T)/cc-pV(T + *d*)Z values (1046 and 1134 cm^−1^, as shown in Table 3) and also show proper intensity behavior. Both $\nu_{3}$ and $\nu_{4}$ show weak satellite band progressions with approximate spacings of 20 cm^−1^ to their blue sides (see inset in Figure 5), which is thought to originate from combination modes with the participation of the Ne-tag (most likely involving the $\omega_{9}$ fundamental (Table 1); see, e.g., Refs. [20,21]). The CH_2_ scissoring mode $\nu_{2}$ is calculated to be very weak and would be located in a region affected by somewhat higher noise and some baseline instabilities. The CH_2_ wagging mode $\nu_{6}$ is calculated to have almost no infrared intensity and is either too weak to be detected or overlapped with the much stronger $\nu_{3}$ band. Apart from that, no other clear spectroscopic feature is observed. As concluded from harmonic fc-CCSD(T)/aug-cc-pV(T + *d*)Z calculations of bare CH_2_Cl^+^ and its singly tagged Ne-clusters (Table 1), tagging is expected to have a negligible influence on the IRPD spectrum of this ion.

### 2.3. IRPD Spectra of CH_2_^37^Cl^+^–Ne and CH_3_^35^ClH^+^–Ne

Because CH_2_^37^Cl^+^–Ne and CH_3_^35^ClH^+^–Ne are isobaric species (m/z=71), both ions were present in the ion trap, and hence, the corresponding spectrum comprises the spectra of both ions superimposed. The center trace in Figure 5 shows the final spectrum obtained in the wavenumber regions from 500 to 1530 cm^−1^ and from 2625 to 2740 cm^−1^. The two regions feature no less than seven vibrational bands, all but one of which can be assigned in a straightforward fashion based on the CCSD(T)/cc-pV(T + *d*)Z force field calculations. The two vibrational bands located at 1037 cm^−1^ and 1125 cm^−1^ are assigned as the strong $\nu_{3}$ and $\nu_{4}$ vibrational fundamentals of CH_2_^37^Cl^+^ (cf. Table 3). By comparison with the results from the CCSD(T)/cc-pV(T + *d*)Z force field calculations collected in Table 4, five bands are assigned as to originate from CH_3_^35^ClH^+^, marking the first spectroscopic detection of this molecular ion. CH_3_ClH^+^ has a total of 3N−6=12 vibrational fundamentals, four of which are lying outside of the spectral regions covered here, namely, the three C–H stretching modes $\nu_{1}$ (calculated at 3085 cm^−1^), $\nu_{2}$ (2979 cm^−1^), and $\nu_{9}$ (3101 cm^−1^), as well as the low-energy torsional mode $\nu_{12}$ (177 cm^−1^). Most other fundamentals are clearly identified, i.e., the Cl–C stretching mode $\nu_{8}$ at 551 cm^−1^, the C–Cl–H bending mode $\nu_{7}$ at 766 cm^−1^, the CH_3_ in-plane rocking mode $\nu_{6}$ at 1083 cm^−1^, and the CH_3_ umbrella mode $\nu_{5}$ at 1345 cm^−1^. According to the calculations, the strong feature at 1420 cm^−1^ is from an overlap of the energetically nearly degenerate CH_3_ asymmetric bending modes $\nu_{4}\left(a^{\prime }\right)$ and $\nu_{10}\left(a^{\prime \prime }\right)$. The vibrational band with the largest calculated infrared intensity (Table 4) and indeed the strongest feature in the spectrum covered here is the Cl–H stretching mode $\nu_{3}$ detected at 2677 cm^−1^. The weak out-of-plane rocking mode $\nu_{11}$ is overlapping with the much stronger $\nu_{3}$ band of CH_2_^37^Cl^+^. Overall, the agreement between the IRPD spectrum and the anharmonic CCSD(T)/cc-pV(T + *d*)Z force field calculation is excellent. The harmonic force field calculations of the bare ions and the singly tagged variants in Table 2 suggest that the influences of Ne should be subtle for most bands but noticeable for some. For the bands covered here, significant shifts might be expected for the $\nu_{3}$ (redshift), $\nu_{7}$ (blueshift), and, possibly, $\nu_{8}$ bands (blueshift) if Ne was connected to the site of protonation. Indeed, the experimental findings are qualitatively consistent with this geometrical arrangement, as the largest differences δ between experimental and calculated wavenumbers of all bands observed are found for these three modes that also agree with the predicted sign of the shift. Lastly, as seen from the inset in Figure 5 (blue center trace), similarly to the $\nu_{3}$ and $\nu_{4}$ bands in CH_2_Cl^+^, the $\nu_{8}$ and $\nu_{7}$ bands (possibly also $\nu_{3}$) show weak satellite bands about 15 cm^−1^ blueshifted from the fundamentals, presumably combination modes involving low-energy fundamentals introduced through Ne-tagging (most likely the $\omega_{15}$ mode, as shown in Table 2).

### 2.4. IRPD Spectrum of CH_3_^37^ClH^+^–Ne

The IRPD spectrum of the m/z=73 species covering the 500 to 1630 cm^−1^ and 2505 to 2880 cm^−1^ regions is shown in Figure 5 (green bottom trace). Clearly, the strong vibrational band quintett comprising the $\nu_{8}$, $\nu_{7}$, $\nu_{5}$, $\nu_{10}/\nu_{4}$, and $\nu_{3}$ bands nicely matches the pattern observed also for the CH_3_^35^ClH^+^–Ne isotopic species at m/z=71. Likewise, the $\nu_{8}$ and $\nu_{7}$ bands of CH_3_^37^ClH^+^–Ne show weaker yet clearly discernible shoulders to their blue sides. IRPD scans of the strong $\nu_{8}$ band reveal that depletion is nearly 100%, suggesting that the m/z=73 spectrum should provide a clean view of the CH_3_^37^ClH^+^–Ne species and not be affected by isobaric contamination. As a consequence, in addition to the weak CH_3_ ip rocking mode $\nu_{6}$ band at 1082 cm^−1^, now also the very weak CH_3_ out-of-plane rocking mode $\nu_{11}$ can be identified at 1023 cm^−1^. In addition to these vibrational fundamentals, the spectrum shows a spectroscopic feature at 1461 cm^−1^ that is also seen as a somewhat less prominent signal in the m/z=71 spectrum (see bands marked with asterisks in Figure 5). From the anharmonic force field calculations, this feature is not compatible with a binary but, possibly, a ternary combination mode comprising the three energetically lowest fundamental modes, $\nu_{7}+\nu_{8}+\nu_{12}$. However, from the present investigation, a definitive assignment is not feasible.

## 3. Materials and Methods

### 3.1. Quantum-Chemical Calculations

Theoretical molecular structures of both CH_2_Cl^+^ ($C_{2v}$) and CH_3_ClH^+^ ($C_{s}$) have previously been provided on several occasions (e.g., Refs. [12,22,23,24,25]). In the present study, complementary quantum-chemical calculations of CH_2_Cl^+^ and CH_3_ClH^+^ (as well as their isostructural and isoelectronic sulfur analogs, H_2_CS and CH_3_SH) were performed at the coupled cluster single and double (CCSD) level augmented by a perturbative treatment of triple excitations (CCSD(T)) [26], together with correlation-consistent (augmented) polarized valence and (augmented) polarized weighted core–valence basis sets, i.e., cc-pV*X*Z [27], aug-cc-pV*X*Z [27,28,29], and cc-pwCV*X*Z [27,30] (with *X* = T, Q). For basis sets denoted as cc-pV(*X* + *d*)Z or aug-cc-pV(*X* + *d*)Z, an additional tight *d* function [31] was added to the chlorine (and sulfur) atom only, while for all other elements, cc-pV(*X*)Z or aug-cc-pV(*X*)Z was used, respectively. Equilibrium geometries were calculated using analytic gradient techniques [32], while harmonic frequencies were computed using analytic second-derivative techniques [33,34]. For anharmonic computations, second-order vibrational perturbation theory (VPT2) [35] was employed, and an additional numerical differentiation of analytic second derivatives was applied to obtain the third and fourth derivatives required for the application of VPT2 [34,36]. The frozen core approximation is indicated throughout with “fc”, and “ae” indicates that all electrons were used in the correlation treatment. All calculations were carried out using the CFOUR program package [37,38].

### 3.2. Experiment

Experimental infrared action spectroscopic characterization of CH_2_Cl^+^ and CH_3_ClH^+^ was performed in selected wavenumber ranges from 500 to 2900 cm^−1^ using a cryogenic 22-pole ion trap apparatus FELion connected to the Free Electron Laser for Infrared eXperiments (FELIX) [39], located at Radboud University (Nijmegen, the Netherlands). The FELion apparatus and its central part, the 22-pole ion trap, have been described elsewhere in detail [40,41], and the experimental conditions applied were similar to those used in other recent studies of molecular ions [16,17,42,43,44]. CH_2_^35,37^Cl^+^ (m/z=49,51) and CH_3_^35,37^ClH^+^ (m/z=51,53) were produced in an ion source under the same experimental conditions from commercially available methyl chloride, CH_3_Cl, using electron impact ionization at an electron energy of about 30 eV. Because of the high natural abundance of ^37^Cl (24.24%), the corresponding heavy isotopologs CH_2_^37^Cl^+^ and CH_3_^37^ClH^+^ were easily accessible. It should be stressed that CH_2_^37^Cl^+^ and CH_3_^35^ClH^+^ are isobaric species (m/z=51); hence, the corresponding spectra were expected to appear superimposed in the same scan.

**Figure A1:**
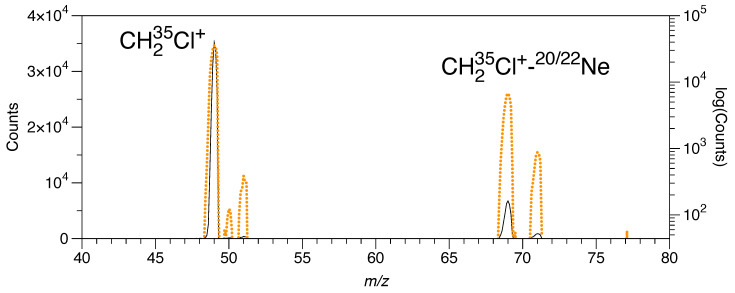
Trap content extracted after initial mass selection for m/z=49, trapping and cooling in the 8 K He/Ne-bath of the 22-pole ion trap featuring some 35,000 CH_2_Cl^+^ ions and almost 7000 weakly bound clusters with ^20^Ne available for IRPD. Black trace and left ordinate: linear plot of counts; orange dotted trace and right ordinate: logarithmic plot of counts.

After extracting the ion cloud from the source and selection in the first quadrupole mass analyzer, the m/z of choice was introduced into the 22-pole ion trap, where it was cooled via a cold pulse of a 3:1 mixture of He:Ne kept at a nominal temperature of 8 K and at a high number density, leading to efficient tagging with Ne atoms. Per filling cycle of the ion trap, typically, several thousand cation–Ne complexes (CH_2_^35,37^Cl^+^–Ne at m/z=69/71 or CH_3_^35,37^ClH^+^–Ne at m/z=71,73) can be formed via three-body collisions (Figure A1 in Appendix C). After a selected storage time of typically 1.6 or 2.6 s, the trap content was extracted from the trap, mass-filtered in a second quadrupole mass analyzer, and counted using a very sensitive Daly-type detector.

In the infrared photodissociation (IRPD) action spectroscopy scheme employed herein, the number of singly tagged CH_2_Cl^+^-Ne cluster ions (similar for CH_3_ClH^+^-Ne) was monitored while the FELIX (FEL-2) IR radiation traversing the ion trap was tuned in wavenumber. FELIX was operated in a pulsed mode (10 Hz), with typical pulse energies of a few up to a few tens of mJ (measured at the exit of the ion trap) and a Fourier-limited full width at half maximum (FWHM) bandwidth on the order of 0.7%. When a vibrational mode of the cluster and the radiation source are coincident in wavenumber, dissociation of the cluster occurs, resulting in depletion in the ion–Ne counts. Finally, several power-normalized spectra were co-added to obtain the final spectrum. Details on the normalization procedure have been given in Ref. [41].

Since the weakly bound, rare gas Ne generally only slightly perturbs the structure of the ion, an IRPD spectrum is highly representative of that of the bare ion (e.g., Refs. [16,17,21,44]). Band parameters such as positions and FWHM were obtained by fitting (multi-component) Gaussian profiles to the experimental spectra. Typical wavenumber uncertainties of 0.5% are mainly dependent on calibration uncertainties due to the grating spectrum analyzer.

## 4. Conclusions

The present study reports on the first infrared gas-phase study of CH_2_Cl^+^ and the first spectroscopic detection and characterization of CH_3_ClH^+^, two fundamental chlorine-bearing ions possibly linked to the astrochemical gas-phase production of methyl chloride, CH_3_Cl. Now that the IR spectra of CH_2_Cl^+^ and CH_3_ClH^+^ have been detected at low spectral resolution, corresponding studies at high resolution are imperative, in particular, to permit radio astronomical searches. Following the very recent technical developments in action spectroscopy of molecular ions, high-resolution infrared spectroscopy of both species may be performed using leak-out spectroscopy [45], a new method that does not require any rare gas tagging and has already been shown, on several occasions, to yield spectra of superb signal-to-noise ratios [46,47,48,49]. The method may even be combined with millimeter-wave radiation in a double-resonance fashion to derive accurate information about the pure rotational spectrum [46,47,50], a prerequisite for radio astronomy. Using the high-level CCSD(T) structural and force field calculations performed here, very good predictions of the ground state rotational and centrifugal distortion constants can be derived. These estimates may even be further improved using a simple scaling procedure, if experimental data of isostructural/isoelectronic species are available (see, e.g., Refs. [16,50,51]). As the Cl^+^ ion is isoelectronic with neutral atomic S, thioformaldehyde, H_2_CS, and methyl mercaptan, CH_3_SH, may potentially be used as calibrators, the pure rotational spectra of which are very well known from previous microwave and millimeter-wave studies (see, e.g., Refs. [52,53] for recent reports). Calculated and experimental rotational constants of H_2_CS and CH_3_SH (structural parameters are given in Appendix B) as well as calculated constants of CH_2_Cl^+^ and CH_3_ClH^+^ are collected in Table 5. For the sulfur species, the agreement between the calculated and experimental ground state rotational constants is already very good. Consequently, the scaling factors, i.e., the ratios $A_{0,exp}$/$A_{0,calc}$, $B_{0,exp}$/$B_{0,calc}$, and $C_{0,exp}$/$C_{0,calc}$ are almost but not quite unity. Multiplying those factors with the calculated ground state rotational constants of CH_2_Cl^+^ and CH_3_ClH^+^ finally yields the scaled best-estimate rotational constants in the last column of Table 5. Both species are very polar with center-of-mass frame equilibrium dipole moment components $\mu_{a}=3.02$ D of CH_2_Cl^+^ as well as $\mu_{a}=1.22$ D and $\mu_{b}=1.42$ D of CH_3_ClH^+^.

From a chemical viewpoint, extending the spectroscopy of CH_2_X^+^ and CH_3_XH^+^ to halogens other than chlorine also seems appealing. The situation for X=Br, for example, appears similar to the one with X=Cl prior to the present study. To date, spectroscopic studies of CH_2_Br^+^ have been restricted to infrared matrix isolation [12,54]. Protonated methyl bromide, CH_3_BrH^+^, seems not to have been spectroscopically studied to date.

## Figures and Tables

**Table 1:** Harmonic vibrational wavenumbers of CH_2_^35^Cl^+^ and CH_2_^35^Cl^+^–Ne calculated at the CCSD(T)/aug-cc-pV(T + *d*)Z level of theory (in cm^−1^) ^1^.

Mode	Bare Ion	Ne–ip	Ne–oop
$\omega_{1}$ CH sym stretching	3102 ($a_{1}$, 18)	3106 ($a^{\prime }$, 17)	3105 ($a^{\prime }$, 16)
$\omega_{2}$ CH_2_ scissoring	1478 ($a_{1}$, 0.3)	1476 ($a^{\prime }$, 1)	1478 ($a^{\prime }$, 0.2)
$\omega_{3}$ Cl–C stretching	1059 ($a_{1}$, 80)	1059 ($a^{\prime }$, 78)	1058 ($a^{\prime }$, 82)
$\omega_{4}$ oop bending	1137 ($b_{1}$, 22)	1138 ($a^{\prime \prime }$, 21)	1136 ($a^{\prime }$, 20)
$\omega_{5}$ CH asym stretching	3239 ($b_{2}$, 51)	3243 ($a^{\prime }$, 59)	3243 ($a^{\prime \prime }$, 50)
$\omega_{6}$ CH_2_ wagging	1047 ($b_{2}$, 0.02)	1048 ($a^{\prime }$, 0.3)	1047 ($a^{\prime \prime }$, 0.003)
$\omega_{7}$ ^2,3^	⋯	93 ($a^{\prime }$, 21)	131 ($a^{\prime \prime }$, 1)
$\omega_{8}$ ^2,4^	⋯	70 ($a^{\prime \prime }$, 0.2)	89 ($a^{\prime }$, 22)
$\omega_{9}$ ^2,5^	⋯	25 ($a^{\prime }$, 1)	34 ($a^{\prime }$, 4)

^1^ Mode symmetries and infrared band intensities (km/mol) are given in parentheses. Mode indices of the Ne-tagged variants are borrowed from bare CH_2_Cl^+^ for the sake of comparability. ^2^ Extra-low-frequency vibrational modes introduced through Ne-tag, arbitrary mode index. ^3^ H–Ne stretching (Ne-ip species) and CH_2_ twisting (Ne-oop species). ^4^ C–H–Ne stretching oop bending (Ne-ip species) and C–Ne stretching (Ne-oop species). ^5^ C–H–Ne ip bending (Ne-ip species) and Cl–C–Ne bending (Ne-oop species).

**Table 2:** Harmonic vibrational wavenumbers of CH_3_^35^ClH^+^ and two CH_3_^35^ClH^+^–Ne variants calculated at the CCSD(T)/aug-cc-pV(T + *d*)Z level of theory (in cm^−1^) ^1^.

Mode	Bare Ion	p-bound	CH_3_/Cl-bound
$\omega_{1}$ C–H asym stretching	3220 ($a^{\prime }$, 18)	3220 ($a^{\prime }$, 17)	3224 ($a^{\prime }$, 20)
$\omega_{2}$ C–H sym stretching	3087 ($a^{\prime }$, 5)	3087 ($a^{\prime }$, 5)	3089 ($a^{\prime }$, 5)
$\omega_{3}$ Cl–H stretching	2823 ($a^{\prime }$, 176)	2811 ($a^{\prime }$, 307)	2824 ($a^{\prime }$, 174)
$\omega_{4}$ CH_3_ asym bending	1472 ($a^{\prime }$, 14)	1472 ($a^{\prime }$, 14)	1471 ($a^{\prime }$, 13)
$\omega_{5}$ CH_3_ umbrella	1386 ($a^{\prime }$, 11)	1386 ($a^{\prime }$, 11)	1385 ($a^{\prime }$, 10)
$\omega_{6}$ CH_3_ ip rocking	1118 ($a^{\prime }$, 3)	1120 ($a^{\prime }$, 3)	1118 ($a^{\prime }$, 3)
$\omega_{7}$ C-Cl-H bending	771 ($a^{\prime }$, 13)	784 ($a^{\prime }$, 11)	773 ($a^{\prime }$, 14)
$\omega_{8}$ Cl–C stretching	565 ($a^{\prime }$, 51)	570 ($a^{\prime }$, 50)	566 ($a^{\prime }$, 50)
$\omega_{9}$ C–H asym stretching	3238 ($a^{\prime \prime }$, 15)	3237 ($a^{\prime \prime }$, 15)	3238 ($a^{\prime \prime }$, 15)
$\omega_{10}$ CH_3_ asym bending	1468 ($a^{\prime \prime }$, 14)	1469 ($a^{\prime \prime }$, 14)	1470 ($a^{\prime \prime }$, 12)
$\omega_{11}$ CH_3_ oop rocking	1040 ($a^{\prime \prime }$, 0.5)	1040 ($a^{\prime \prime }$, 0.5)	1040 ($a^{\prime \prime }$, 0.4)
$\omega_{12}$ torsion	202 ($a^{\prime \prime }$, 39)	244 ($a^{\prime \prime }$, 43)	211 ($a^{\prime \prime }$, 42)
$\omega_{13}$ ^2,3^	⋯	90 ($a^{\prime }$, 7)	69 ($a^{\prime }$, 8)
$\omega_{14}$ ^2,4^	⋯	72 ($a^{\prime \prime }$, 7)	46 ($a^{\prime }$, 0.4)
$\omega_{15}$ ^2,5^	⋯	20 ($a^{\prime }$, 7)	40 ($a^{\prime \prime }$, 10)

^1^ Mode symmetries and infrared band intensities (km/mol) are given in parentheses. ^2^ Extra-low-frequency vibrational modes introduced through Ne-tag, arbitrary mode index. ^3^ H–Ne stretching. ^4^ Ne–H–Cl oop bending (p-bound species) and Ne–H–Cl ip bending (CH_3_/Cl-bound species). ^5^ Ne–H–Cl ip bending (p-bound species) and Ne–H–Cl oop bending (CH_3_/Cl-bound species).

**Table 3:** Harmonic and anharmonic fc-CCSD(T)/cc-pV(T + *d*)Z vibrational wavenumbers (in cm^−1^) and infrared band intensities (int, km/mol), experimental wavenumbers (exp, cm^−1^), full width at half maximum of observed bands (FWHM, cm^−1^), and difference between observed and calculated anharmonic wavenumbers δ (cm^−1^) of CH_2_Cl^+^.

**Mode**	**CH_2_^35^Cl^+^**					
	**Harm**	**Anharm**	**Int**	**Exp ^1^**	**FWHM**	δ
$\nu_{1}\left(a_{1}\right)$ CH sym stretching	3105.2	2993.3	14	nc	⋯	⋯
$\nu_{2}\left(a_{1}\right)$ CH_2_ scissoring	1491.1	1451.1	0.7	⋯	⋯	⋯
$\nu_{3}\left(a_{1}\right)$ Cl–C stretching	1061.8	1045.6	81	1048	13	2
$\nu_{4}\left(b_{1}\right)$ oop bending	1151.9	1134.1	27	1129	17	−5
$\nu_{5}\left(b_{2}\right)$ CH asym stretching	3243.9	3101.1	47	nc	⋯	⋯
$\nu_{6}\left(b_{2}\right)$ CH_2_ wagging	1054.8	1038.2	<0.01	⋯	⋯	⋯
**Mode**	**CH_2_^37^Cl^+^**					
	**Harm**	**Anharm**	**Int**	**Exp ^1^**	**FWHM**	δ
$\nu_{1}\left(a_{1}\right)$ CH sym stretching	3105.2	2993.3	14	nc	⋯	⋯
$\nu_{2}\left(a_{1}\right)$ CH_2_ scissoring	1491.0	1451.0	0.7	⋯	⋯	⋯
$\nu_{3}\left(a_{1}\right)$ Cl–C stretching	1053.6	1037.6	82	1037	15	−1
$\nu_{4}\left(b_{1}\right)$ oop bending	1151.8	1134.0	27	1125	13	−9
$\nu_{5}\left(b_{2}\right)$ CH asym stretching	3243.9	3101.1	47	nc	⋯	⋯
$\nu_{6}\left(b_{2}\right)$ CH_2_ wagging	1054.2	1037.6	<0.01	⋯	⋯	⋯

^1^ Wavenumber errors amount to 0.5% of the band position; nc: not covered.

**Table 4:** Harmonic and anharmonic fc-CCSD(T)/cc-pV(T + *d*)Z vibrational wavenumbers (in cm^−1^) and infrared band intensities (int, km/mol), experimental wavenumbers (exp, cm^−1^), full width at half maximum of observed bands (FWHM, cm^−1^), and difference between observed and calculated wavenumbers δ (cm^−1^) of CH_3_ClH^+^.

**Mode**	**CH_3_^35^ClH^+^**					
	**Harm**	**Anharm**	**Int**	**Exp** ^1^	**FWHM**	δ
$\nu_{1}\left(a^{\prime }\right)$ C–H asym stretching	3225.4	3084.9	16	nc	⋯	⋯
$\nu_{2}\left(a^{\prime }\right)$ C–H sym stretching	3091.6	2978.8	5	nc	⋯	⋯
$\nu_{3}\left(a^{\prime }\right)$ Cl–H stretching	2829.5	2713.5	173	2677	15	−37
$\nu_{4}\left(a^{\prime }\right)$ CH_3_ asym bending	1465.1	1420.6	15	1415	10	−6
$\nu_{5}\left(a^{\prime }\right)$ CH_3_ umbrella	1378.2	1348.1	10	1345	8	−3
$\nu_{6}\left(a^{\prime }\right)$ CH_3_ ip rocking	1113.6	1080.8	3	1083	9	2
$\nu_{7}\left(a^{\prime }\right)$ C-Cl-H bending	768.4	757.1	14	766	9	9
$\nu_{8}\left(a^{\prime }\right)$ Cl–C stretching	569.8	544.1	55	551	11	7
$\nu_{9}\left(a^{\prime \prime }\right)$ C–H asym stretching	3242.9	3100.6	14	nc	⋯	⋯
$\nu_{10}\left(a^{\prime \prime }\right)$ CH_3_ asym bending	1462.5	1419.6	15	1415	10	−5
$\nu_{11}\left(a^{\prime \prime }\right)$ CH_3_ oop rocking	1033.2	1020.6	1	blended	⋯	⋯
$\nu_{12}\left(a^{\prime \prime }\right)$ torsion	205.5	177.4	45	nc	⋯	⋯
**Mode**	**CH_3_^37^ClH^+^**					
	**Harm**	**Anharm**	**Int**	**Exp** ^1^	**FWHM**	** δ **
$\nu_{1}\left(a^{\prime }\right)$ C–H asym stretching	3225.4	3084.9	16	nc	⋯	⋯
$\nu_{2}\left(a^{\prime }\right)$ C–H sym stretching	3091.6	2978.8	5	nc	⋯	⋯
$\nu_{3}\left(a^{\prime }\right)$ Cl–H stretching	2827.3	2711.5	173	2678	20	−34
$\nu_{4}\left(a^{\prime }\right)$ CH_3_ asym bending	1465.1	1420.5	15	1416	13	−5
$\nu_{5}\left(a^{\prime }\right)$ CH_3_ umbrella	1377.9	1347.9	10	1344	11	−4
$\nu_{6}\left(a^{\prime }\right)$ CH_3_ ip rocking	1112.7	1079.9	3	1082	11	2
$\nu_{7}\left(a^{\prime }\right)$ C-Cl-H bending	768.1	756.8	14	765	9	8
$\nu_{8}\left(a^{\prime }\right)$ Cl–C stretching	565.3	539.9	56	547	11	7
$\nu_{9}\left(a^{\prime \prime }\right)$ C–H asym stretching	3242.9	3100.6	14	nc	⋯	⋯
$\nu_{10}\left(a^{\prime \prime }\right)$ CH_3_ asym bending	1462.5	1419.6	15	1416	13	−4
$\nu_{11}\left(a^{\prime \prime }\right)$ CH_3_ oop rocking	1032.8	1020.2	1	1023	12	3
$\nu_{12}\left(a^{\prime \prime }\right)$ torsion	205.4	177.3	45	nc	⋯	⋯

^1^ Wavenumber errors amount to 0.5% of the band position; nc: not covered.

**Table 5:** Rotational parameters of H_2_CS, CH_2_^35^Cl^+^, CH_3_SH, and CH_3_^35^ClH^+^ (in MHz) ^1^.

**Parameter**	**H_2_CS**		**CH_2_^35^Cl^+^**	
	**Calc.**	**Exp. [52]**	**Calc.**	**Scaled**
$A_{e}$	295,043.772	⋯	278,232.178	⋯
$B_{e}$	17,770.906	⋯	17,929.887	⋯
$C_{e}$	16,761.346	⋯	16,844.397	⋯
$\Delta A_{0}$	1928.402	⋯	2249.656	⋯
$\Delta B_{0}$	76.366	⋯	91.765	⋯
$\Delta C_{0}$	109.739	⋯	125.604	⋯
$A_{0}$	293,115.370	291,613.339	275,982.522	274,568.285
$B_{0}$	17,694.540	17,698.994	17,838.122	17,842.613
$C_{0}$	16,651.607	16,652.498	16,718.793	16,719.687
**Parameter**	**CH_3_SH**		**CH_3_^35^ClH^+^**	
	**Calc**.	**Exp.** [53] ^2^	**Calc.**	**Scaled**
$A_{e}$	103,863.953	⋯	103,144.040	⋯
$B_{e}$	13,029.847	⋯	12,472.858	⋯
$C_{e}$	12,493.723	⋯	12,016.948	⋯
$\Delta A_{0}$	1054.186	⋯	1069.132	⋯
$\Delta B_{0}$	131.546	⋯	207.375	⋯
$\Delta C_{0}$	117.267	⋯	184.225	⋯
$A_{0}$	102,809.767	102,767.147	102,074.907	102,032.592
$B_{0}$	12,898.301	12,951.372	12,265.483	12,315.950
$C_{0}$	12,376.457	12,388.033	11,832.724	11,843.792

^1^ Equilibrium rotational constants and zero-point vibrational contributions calculated at the ae-CCSD(T)/cc-pwCVQZ and fc-CCSD(T)/cc-pV(T + *d*)Z levels of theory, respectively. ^2^ For the sake of comparability, the rotational constants from Ref. [53] given in the rho-axis system were converted to the principal axis system. Differences are virtually negligible.

## Data Availability

Data are contained within the article.

## Appendix A. Molecular Structures of CH_2_Cl^+^ and CH_3_ClH^+^ and Their Weakly Bound Clusters with Neon

### Appendix A.1. CH_2_Cl^+^

CL

C 1 r1

H 2 r2 1 a1

H 2 r2 1 a1 3 d180

       cc-pV(T+d)Z / aug-cc-pV(T+d)Z / cc-pwCVQZ

r1   =   1.59619   /   1.59662       /   1.58812

r2   =   1.08829   /   1.08860       /   1.08620

a1   = 119.09851   / 119.03255       / 119.07897

d180 = 180.00000   / 180.00000       / 180.00000

### Appendix A.2. CH_2_Cl^+^ Ne-ip

CL

C 1 r1

H 2 r2 1 a1

H 2 r3 1 a2 3 d180

NE 4 r4 2 a3 3 d180

      aug-cc-pV(T+d)Z

r1   =    1.59672

r2   =    1.08853

a1   =  119.01368

r3   =    1.08827

a2   =  118.82770

d180 =  180.00000

r4   =    2.39056

a3   =  133.32909

### Appendix A.3. CH_2_Cl^+^ Ne-oop

CL

C 1 r1

NE 2 rNE 1 aNE

H 2 r2 3 a1 1 d1

H 2 r2 3 a1 1 md1

      aug-cc-pV(T+d)Z

r1   =    1.59691

rNE  =    2.79264

aNE  =  108.25118

r2   =    1.08829

a1   =   81.35078

d1   =  117.85276

md1  = −117.85276

### Appendix A.4. CH_3_ClH^+^

H

C 1 r1

CL 2 r2 1 a1

H 3 r3 2 a2 1 d180

H 2 r4 3 a3 1 d1

H 2 r4 3 a3 1 md1

       cc-pV(T+d)Z / aug-cc-pV(T+d)Z / cc-pwCVQZ

r1   =    1.08652  /    1.08729     /    1.08435

r2   =    1.85970  /    1.86174     /    1.84696

a1   =  101.97978  /  101.91486     /  102.10778

r3   =    1.29569  /    1.29687     /    1.29436

a2   =   99.43628  /   99.36597     /   99.64973

d180 =  180.00000  /  180.00000     /  180.00000

r4   =    1.08443  /    1.08514     /    1.08215

a3   =  105.70626  /  105.53383     /  105.84821

d1   =  118.70156  /  118.73149     /  118.68039

md1  = −118.70156  / −118.73149     / −118.68039

### Appendix A.5. CH_3_ClH^+^–Ne, p-bound

H

C 1 r1

CL 2 r2 1 a1

H 3 r3 2 a2 1 d180

H 2 r4 3 a3 1 d1

H 2 r4 3 a3 1 md1

X 4 rd 3 a90 2 d180

NE 4 r5 7 a4 3 d180

r1   =        1.08720

r2   =        1.85987

a1   =      102.05144

r3   =        1.29827

a2   =       99.19249

d180 =      180.00000

r4   =        1.08515

a3   =      105.57033

d1   =      118.75594

md1  =     −118.75594

rd   =        1.00000

a90  =       90.00000

r5   =        2.07322

a4   =       94.38835

### Appendix A.6. CH_3_ClH^+^–Ne, CH3/Cl-bound

H

C 1 r1

CL 2 r2 1 a1

H 3 r3 2 a2 1 d180

H 2 r4 3 a3 1 d1

H 2 r4 3 a3 1 md1

X 1 rd 2 a90 3 d0

NE 1 r5 7 a4 2 d180

r1   =        1.08692

r2   =        1.86155

a1   =      101.81875

r3   =        1.29674

a2   =       99.27846

d180 =      180.00000

r4   =        1.08514

a3   =      105.52239

d1   =      118.75328

md1  =     −118.75328

rd   =        1.00000

a90  =       90.00000

d0   =        0.00000

r5   =        2.57934

a4   =       40.24330

## Appendix B. Molecular Structures of H_2_CS and CH_3_SH

### Appendix B.1. H_2_CS

S

C 1 r1

H 2 r2 1 a1

H 2 r2 1 a1 3 d180

            cc-pV(T+d)Z / cc-pwCVQZ

r1   =          1.61826 /   1.60890

r2   =          1.08766 /   1.08531

a1   =        121.92777 / 121.85524

d180 =        180.00000 / 180.00000

### Appendix B.2. CH_3_SH

H

C 1 r1

S 2 r2 1 a1

H 3 r3 2 a2 1 d180

H 2 r4 3 a3 1 d1

H 2 r4 3 a3 1 md1

            cc-pV(T+d)Z /  cc-pwCVQZ

r1   =          1.08915 /    1.08669

r2   =          1.82085 /    1.81020

a1   =        106.24841 /  106.25194

r3   =          1.33803 /    1.33502

a2   =         96.64050 /   96.88414

d180 =        180.00000 /  180.00000

r4   =          1.08804 /    1.08554

a3   =        111.27155 /  111.25848

d1   =        118.26139 /  118.25357

md1  =       −118.26139 / −118.25357
